# Buschke-Loewenstein Tumor: A Case Report and Advocacy for Human Papillomavirus Vaccination

**DOI:** 10.7759/cureus.10789

**Published:** 2020-10-04

**Authors:** Lara E Saikaly, Sami K Saikaly, Robert Norman

**Affiliations:** 1 Epidemiology, University of Florida College of Public Health and Health Professions, Gainesville, USA; 2 Dermatology, University of Central Florida College of Medicine, Orlando, USA; 3 Internal Medicine, University of Central Florida College of Medicine, Orlando, USA

**Keywords:** human papillomavirus, vaccination, sexually transmitted diseases, buschke-loewenstein tumor, verrucous carcinoma, preventive medicine

## Abstract

Human papillomavirus (HPV) is a widespread sexually transmitted infection which can lead to genital warts, squamous cell carcinoma, and verrucous carcinomas. Buschke-Loewenstein tumor (BLT) is a verrucous carcinoma which occurs in the anogenital and perianal areas. BLT is often associated with HPV 6 and 11, but HPV is preventable through routine vaccination. The Centers for Disease Control and Prevention (CDC) provides recommendations for routine HPV vaccination among both adolescents and adults. Following these guidelines may result in decreased incidence of BLT and minimize the need for subsequent invasive treatment. Here we present a case of BLT and advocate the use of HPV vaccination among patients to prevent discussed potential adverse outcomes.

## Introduction

Human papillomavirus (HPV), a widespread sexually transmitted infection most known for its role in cervical cancer, also plays a role in genital tumors in males. This virus can lead to many clinical presentations, from warts to squamous cell carcinoma [1]. It is highly preventable through vaccination for both adolescents and adults, and the Centers for Disease Control and Prevention (CDC) recommends vaccination for all individuals up to 26 years old. Routine vaccination is recommended for adolescents starting at 11 or 12 years of age [2]. A less frequent manifestation of HPV is the Buschke-Loewenstein tumor (BLT). BLT is a verrucous carcinoma with a high risk of recurrence. Treatment usually requires aggressive surgical removal as BLT is locally aggressive. Here we present a case of a large BLT which highlights a serious HPV outcome and the importance of vaccination for this potentially preventable communicable disease.

## Case presentation

**Figure 1:**
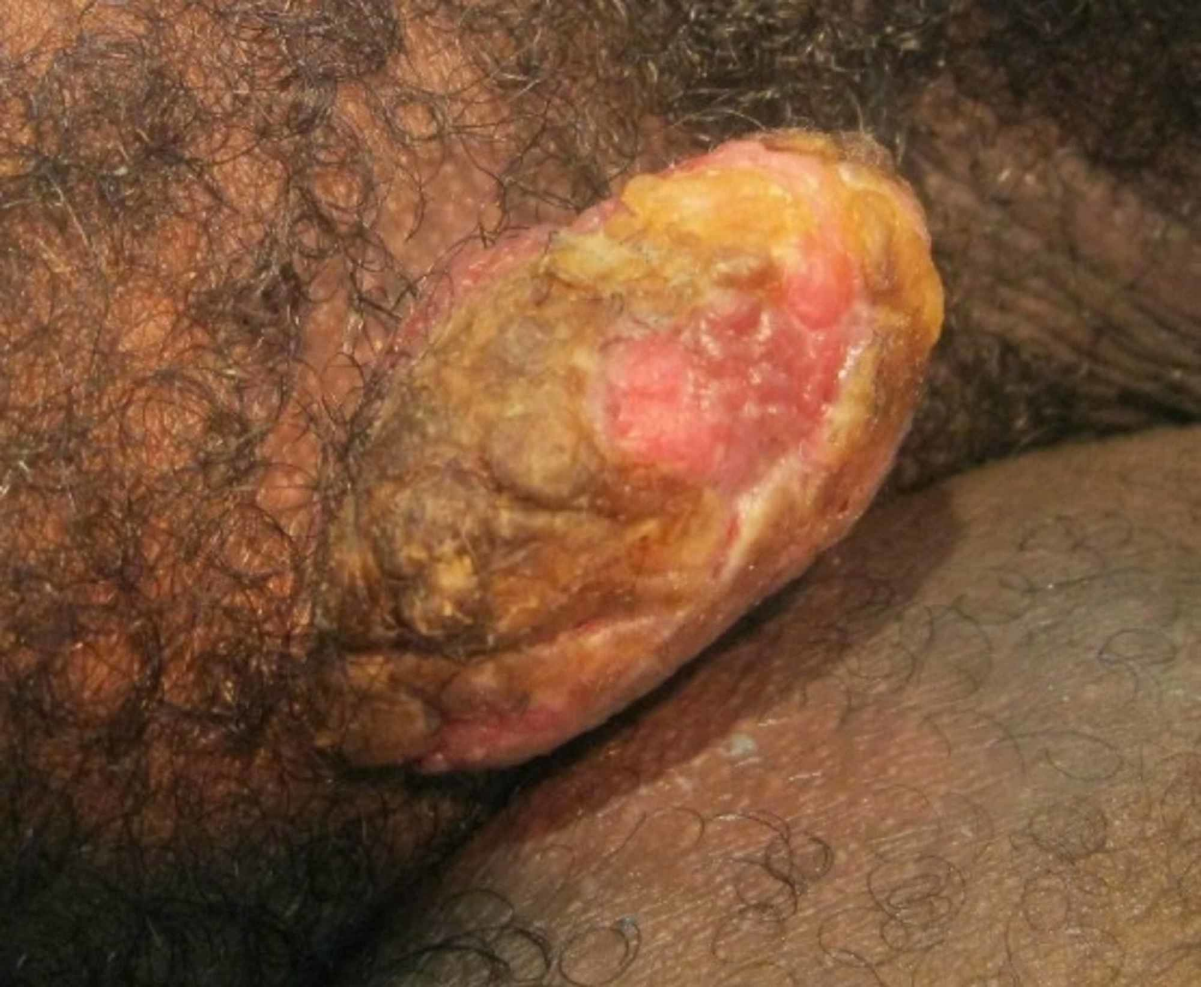
Large pink, yellow protruding plaque in R groin

**Figure 2:**
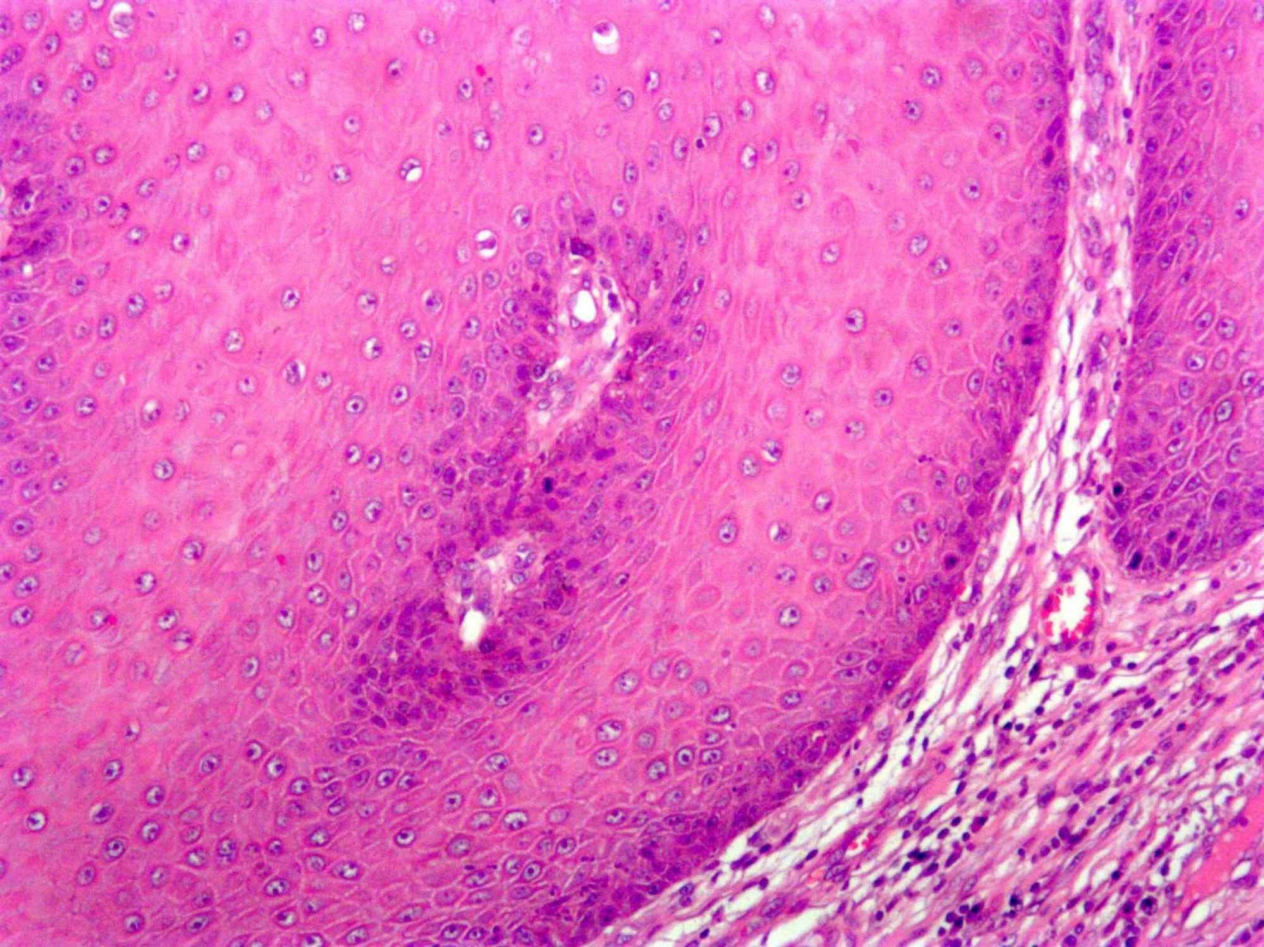
Atypical glassy squamous cells with mild nuclear atypia, koilocytic cell changes, and inflammatory infiltrate (10x, hematoxylin and eosin stain)

A 44-year-old African American male with no relevant past medical history presented to the dermatology clinic with the chief complaint of a bothersome large growth in his right groin which had been present for one year. On physical exam, a 3.5 x 2.5 x 2.0 cm pink protuberant plaque was evident (Figure 1). An elliptical excision was performed with 2.7 cm margins down to subcutaneous tissue. Histology showed broad rete ridges with smooth, bulbous, pushing borders into the dermis, with hyperkeratosis, papillomatosis, and acanthotic epidermis. The epidermis was comprised of atypical glassy squamous cells with mild nuclear atypia, koilocytic cell change, and inflammatory infiltrate (Figure 2). These findings are suggestive of giant condyloma of Buschke and Loewenstein, a verrucous carcinoma. The patient was then referred to a general surgeon to ensure clear margins. No other treatments were attempted prior to referral. After referral to general surgery, the patient was lost to follow-up.

## Discussion

BLT, also called "giant condyloma acuminatum", is a verrucous carcinoma which occurs in the anogenital and perianal areas [3-4]. The differential diagnosis includes condyloma acuminatum [5]. BLT has a large infiltrative base and is slow growing, locally aggressive, and disfiguring [3,5-6]. Histologically, epidermal hyperplasia, hyperkeratosis, and koilocytosis are seen [7].

While it rarely metastasizes [3], BLT has a high risk of recurrence and carries the chance of malignant conversion [6], even within a condyloma acuminatum [4]. Most BLT cases are associated with HPV 6 and 11, with a few cases involving the highly oncogenic HPV 16 and 18 [3,5-6]. Other related factors include not being circumcised, poor hygiene, chronic irritation, human immunodeficiency virus (HIV) or human T-lymphotropic virus type I (HTLV-I) immunosuppression, recurrent genital warts, and sexual promiscuity [6].　

Initial treatment is commonly surgical excision [4]. While simple excision has been reported as sufficient [4], aggressive treatment via wide surgical excision is preferred [4-5]. Skin grafting can be performed after the excision [5-6]. Given the tumor location, genital reconstruction may be necessary. If surgical intervention is declined, chemotherapeutic management with systemic agents such as 5-fluorouracil, cisplatin, and mitomycin-based medications may be utilized [3-4]. Intralesional 5-fluorouracil has also proven effective. Finally, laser ablation therapy, local application of 5% imiquimod cream, and intralesional interferon are other treatment options [4].

From an epidemiologic viewpoint, proper vaccination would minimize BLT disease burden and subsequent potentially invasive treatment. Vaccine herd effects indirectly provide protection to unvaccinated individuals, but these individuals still remain susceptible to infection [8]. The HPV quadrivalent vaccine (HPV4; Gardasil (Merck, Whitehouse Station, NJ)) is of interest as it targets HPV 6, 11, 16, and 18 [9]. One study found that vaccination with HPV4 among men showed an effectiveness of 84% in preventing external genital lesions caused by all HPV types and 90% against genital lesions related to HPV 6, 11, 16, and 18 [9]. An HPV 9-valent vaccine (Gardasil 9) was approved for use in 2014 [10] and was recently approved in 2018 by the Food and Drug Administration (FDA) to include individuals between 27 and 45 years old [11].

## Conclusions

BLT is a verrucous carcinoma that is slow growing, locally aggressive, and disfiguring. BLT has a high risk of recurrence, and treatment usually requires aggressive surgical removal. Alternative treatment options include chemotherapeutic management and laser ablation therapy, among others. BLT is often associated with HPV 6 and 11, both of which are preventable infectious diseases. Thus, proper implementation of routine HPV vaccination following the CDC’s guidelines may be an effective preventative measure for BLT among individuals from all age groups. Overall, vaccination of the target population with the HPV quadrivalent vaccine or HPV 9-valent vaccine should be recommended as it minimizes susceptibility to infection, decreases the incidence of novel cases, and decreases the prevalence of BLT.
